# Complete Genome Sequence of a Novel Very Virulent Strain of Infectious Bursal Disease Virus Circulating in Russia

**DOI:** 10.1128/MRA.01084-18

**Published:** 2018-11-21

**Authors:** Dmitriy A. Shirokov, Alexandr S. Dubovoi, Valentin A. Manuvera, Galina N. Samuseva, Margarita E. Dmitrieva, Vassili N. Lazarev

**Affiliations:** a Federal Research and Clinical Center of Physical-Chemical Medicine of Federal Medical Biological Agency, Moscow, Russian Federation; b K. I. Skryabin Moscow State Academy of Veterinary Medicine and Biotechnology, Moscow, Russian Federation; c All-Russian Research Veterinary Institute of Poultry Science, St. Petersburg, Russian Federation; d Moscow Institute of Physics and Technology, Dolgoprudny, Russian Federation; KU Leuven

## Abstract

A novel strain of infectious bursal disease virus, named DD1, was isolated from broiler chickens in Russia in 2016. Here, we present its complete genome sequence. Nucleotide sequence analysis of both segments of the virus suggests that it belongs to a group of very virulent strains.

## Abstract (precis)

A novel strain of infectious bursal disease virus, named DD1, was isolated from broiler chickens in Russia in 2016. Here, we present its complete genome sequence.

## ANNOUNCEMENT

Infectious bursal disease virus (IBDV) represents one of the main problems for the poultry industry. This virus attacks and destroys immature B lymphocytes in the bursa of Fabricius (BF) of young chickens, which leads to severe immunosuppression and, as a result, increased susceptibility of the bird to a wide range of secondary viral and bacterial infections, often resulting in death (1, 2). IBDV belongs to the genus Avibirnavirus of the Birnaviridae family, and its genome consists of two segments of linear double-stranded RNA (A and B). There are 2 IBDV serotypes, but only serotype 1 is pathogenic for chickens. Serotype 1, in turn, is divided into four groups of strains, “classical,” “very virulent,” “variant,” and “vaccine” (3).

Strain DD1 of IBDV was isolated in 2016 from broiler chickens in the Volgograd region of Russia. The BF of chickens with clinical signs were homogenized and exposed to 3 freeze-thaw cycles. The homogenate was centrifuged at 3,000 × *g* for 10 min, and the virus-containing supernatant was filtered through a 0.22-μm filter. The diagnosis was confirmed by the agar gel immunodiffusion test, using a specific serum (4). Experimental infection of 35- to 40-day-old specific-pathogen-free (SPF) chickens with this strain resulted in the death of 80% of the flock (the animal use protocol was reviewed and approved by the ethics committee of the All-Russian Research Veterinary Institute of Poultry Science). The genetic material of the virus was isolated from the homogenized tissues of the BF of affected birds using the ExtractRNA kit (Evrogen). The complete genome sequence was obtained by overlapping reverse transcriptase PCR (RT-PCR) (using RevertAid reverse transcriptase and Phusion high-fidelity DNA polymerase [Thermo Scientific]) and Sanger dideoxy sequencing in both directions. Nucleotide sequences were assembled in Vector NTI 10.3.0, and multiple sequence alignments (using MUltiple Sequence Comparison by Log-Expectation [MUSCLE] and ClustalW algorithms) and phylogenetic tree construction (using the neighbor-joining method) were performed in Ugene 1.30.0 (5). The length of segment A of DD1 is 3,222 bp (with a GC content of 53.97%), and the length of segment B of DD1 is 2,815 bp (with a GC content of 52.50%).

Segment A of IBDV contains 2 overlapping open reading frames encoding a structural polyprotein (further processed on VP2, VP4, and VP3) and the VP5 protein. Sequence analysis of segment A of strain DD1 showed that both the capsid protein VP2 and the VP5 protein have amino acid signatures specific to very virulent strains (6, 7). For VP2, these are the residues A222, I256, I294, and S299, located in the loops of the viral β-barrels, which are present in the immunodominant epitopes (8), and for VP5, the residues are R49 and W137. Segment B encodes the VP1 protein—the RNA-dependent RNA polymerase (RdRp) of the virus. The RdRp of the DD1 strain contains the TDN tripeptide at positions 145 to 147, which is characteristic of very virulent strains of IBDV (9).

**FIG 1:**
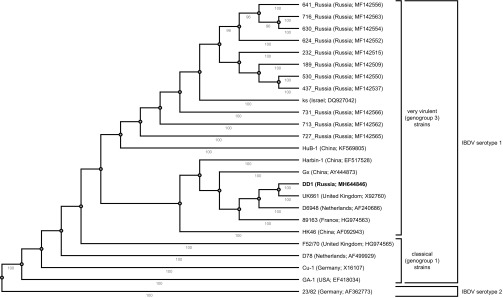
Phylogenetic relationships between some very virulent (genogroup 3) strains of IBDV. The nucleotide sequence encoding the hypervariable region of the VP2 protein (hvVP2) was used as a phylogenetic marker. The analysis was performed in Ugene 1.30.0, using the neighbor-joining method with 100 bootstrap replicates. Only bootstrap values greater than 90 are shown. Four classical strains of IBDV serotype 1 and one strain of IBDV serotype 2 were used as outgroups. The countries of origin of the strains and the GenBank accession numbers for complete segment A (where possible) or hvVP2 sequences are given in brackets after the strain names. Strain DD1 (accession number MH644846), presented in this paper, is shown in bold.

The search for homologous sequences in GenBank and phylogenetic analysis showed that the DD1 strain is closely related to the European very virulent strains, UK661 (United Kingdom), D6948 (Netherlands), and 89163 (France), and to some Chinese strains, namely, HK46, Gx, and Harbin-1 (Fig. 1). According to a new classification for IBDV (10), the DD1 strain belongs to genogroup 3. Interestingly, the DD1 strain differs phylogenetically from Russian strains included in this genogroup. It confirms the great genetic diversity of the IBDV strains circulating in the territories of the largest country in the world. In conclusion, it should be noted that the sequence presented by us is the first complete genome sequence of the IBDV strain from Russia. Until now, only short sequences of segments A and B of Russian strains were available in GenBank (10, 11).

### Data availability.

The genome sequence of DD1 has been deposited in GenBank under the accession numbers MH644846 (segment A) and MH644847 (segment B).
